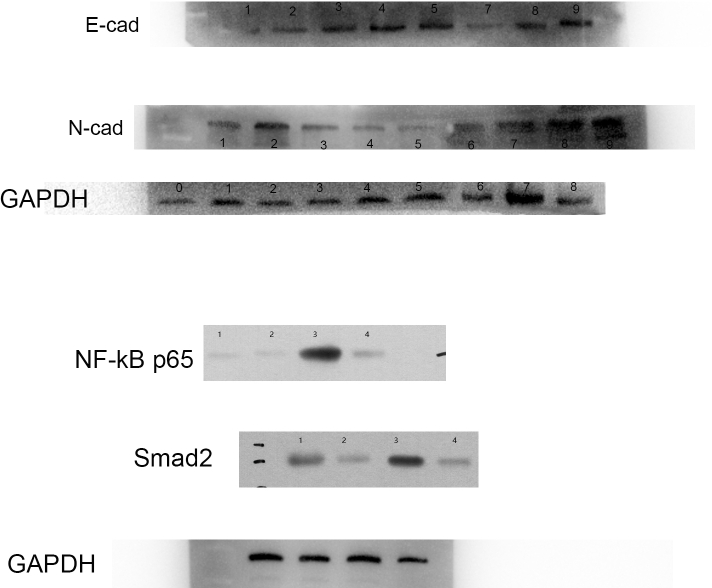# Berberine suppressed the epithelial–mesenchymal transition (EMT) of colon epithelial cells through the TGF-β1/Smad and NF-κB pathways associated with miRNA-1269a

**DOI:** 10.1016/j.heliyon.2024.e36059

**Published:** 2024-08-12

**Authors:** chao Huang, Haosheng liu, Yidian Yang, Yue He, Weizeng shen

**Affiliations:** Department of Traditional Chinese Medicine, The Second Affiliated Hospital of Shenzhen University (People's Hospital of Shenzhen Baoan District), Shenzhen, 518100, China

**Keywords:** Epithelial–mesenchymal transition, Berberine, Colon cancer, Transforming growth factor beta 1, Noncoding RNA

## Abstract

**Objective:**

To explore the mechanisms of the TGF-β1/Smad and NF-κB pathways in the effect of berberine (BBR) on colon cancer epithelial–mesenchymal transition (EMT) and their regulatory relationships with microRNAs (miRNAs).

**Methods:**

TGF-β1 was used to induce EMT in normal colon epithelial HCoEpiC cells and colon cancer HT29 cells in vitro. After BBR intervention, the expression of EMT-related markers and the major molecules involved in the TGF-β1/Smad and NF-κB pathways were detected via western blotting. Cell migration was detected via wound healing assays. SMAD2 and NF-κB p65 were overexpressed and transfected into cells, and the inhibitors SB431542 and BAY 11–7082 were added to block the TGF-β1/Smad and NF-κB pathways, respectively. The mRNA expression levels of related microRNA genes were detected by using RT‒PCR.

**Results:**

Treatment with 10 ng/ml TGF-β1 for 72 h significantly induced EMT in HCoEpiC and HT29 cells, which was repressed by BBR. BBR significantly inhibited the TGF-β1-induced migration of HCoEpiC and HT29 cells and the TGF-β1-promoted expression of p-Smad2/3, NF-κB p65, and p-IκBα. Compared to those in the group treated with TGF-β1, the expression of NF-κB p65 and p-Smad2 in the group treated with NF-κB pathway inhibitor BAY 11–7082 was decreased (*P* < 0.05), and TGF-β1 signalling inhibitor SB431542 significantly reduced the expression of NF-κB p65 (*P* < 0.05). Overexpression of NF-κB p65 and SMAD2 in HT29 cells decreased the expression of *E*-cadherin and caused a relative increase in N-cadherin. BBR mediated the expression profile of microRNAs in TGF-β1-induced HCoEpiC cells, but this pattern differed from that in HT29 cells. SB431542 and BAY 11–7082 significantly reduced the mRNA level of miR-1269a in HCoEpiC and HT29 cells (*P* < 0.05). Overexpressed NF-κB p65 and SMAD2 increased the mRNA level of miR-1269a in both cell lines; however, this increase was significantly lower than that in the TGF-β1 treatment group (*P* < 0.05).

**Conclusion:**

BBR can significantly inhibit TGF-β1-induced EMT in normal and cancerous colon epithelial cells through the inhibition of the TGF-β1/Smad and NF-κB p65 pathways. TGF-β1/Smads can promote the NF-κB p65 pathway, which is a common target of miR-1269a, and can partially regulate the expression of miR-1269a.

## Abbreviation

ANOVA: Analysis of variance
BBR: Berberine
EMT: Epithelial-Mesenchymal Transition
FBS: Fetal Bovine Serum
NF-κB: Nuclear Factor Kappa-light-chain-enhancer of activated B
RIPA: Ristocetin-induced platelet agglutination
R-Smads: Receptor-regulated Smads
RT-PCR: Real-time quantitative polymerase chain reaction
SDS-PAGE: Sodium dodecyl sulfate polyacrylamide gel electrophoresis
SD: Standard deviation
Smad: Drosophila mothers against decapentaplegic
TGF-β1: Transforming growth factor beta 1

## 1 Introduction

Recent research advances have provided new information on the pivotal role of epithelial–mesenchymal transition (EMT) in the progression of colorectal cancer. EMT has been identified as being a critical step for the invasion and metastasis of colorectal cancer. These findings underscore the need for further research into EMT-targeting therapies, which could improve treatment outcomes for patients with colorectal cancer.

Berberine (BBR), which is a natural isoquinoline alkaloid derived from various plants such as Berberis, has been traditionally used in Chinese medicine for its antimicrobial, anti-inflammatory, and anti-diabetic effects [[1], [2], [3], [4]]. Recent research has extended the potential therapeutic applications of BBR to include its role in modulating EMT [5,6]. The modulation of EMT via BBR has garnered attention for its potential in cancer therapy. BBR can inhibit EMT in various cancer cell lines [[7], [8], [9]], including breast, lung, and colorectal cancer cell lines. It exerts its effects through multiple signalling pathways that are critical for the induction of EMT. One of the key mechanisms involves the downregulation of the transforming growth factor beta 1 (TGF-β1)/Smad pathway. BBR has been reported to inhibit TGF-β-induced Smad2/3 phosphorylation [10,11], thereby blocking the nuclear translocation of Smad4 and the subsequent transcription of EMT-related genes.

The critical role of the TGF-β1/Smad signalling pathway in EMT has been increasingly recognized. In particular, the TGF-β1/Smad pathway has garnered attention for its dual role in both tumour suppression and promotion, depending on the cellular context and tumour stage. Upon TGF-β1 stimulation, type I and type II serine/threonine kinase receptors are activated, thus phosphorylating receptor-regulated Smads (R-Smads). Phosphorylated R-Smads then form complexes with Smad4, which translocates into the nucleus to regulate the expression of target genes involved in EMT.

The TGF-β1 and nuclear factor kappa-light-chain-enhancer of activated B (NF-κB) pathways are pivotal in regulating a myriad of cellular processes, including proliferation, apoptosis, inflammation, and immune responses. An understanding of the crosstalk between the TGF-β1 and NF-κB pathways is crucial for deciphering the molecular mechanisms underlying various diseases, including colorectal cancer, and for identifying potential therapeutic targets. NF-κB is a transcription factor that, when activated, translocates to the nucleus and induces the expression of genes involved in inflammation, proliferation, and cell survival. Dysregulation of NF-κB signalling is associated with the development of chronic inflammatory diseases and cancer. BBR has been identified as being a potent inhibitor of NF-κB activation [12], thereby exhibiting anti-inflammatory and anticancer activities.

In previous studies [13,14], we performed gene chip analysis on neonatal rats colon cancer tissues and found that after being stimulated by TGF-β1, a variety of microRNAs (miRNAs) with significant differences, such as miRNA-29a, miR-1269a, miR-135b and miR-21, could be affected by BBR to varying degrees. Recently, BBR has been found to modulate the expression of miRNAs involved in EMT [[15], [16], [17]]. For instance, BBR upregulated the expression of miR-200 family members, which are known to suppress EMT by targeting the transcription factors ZEB1 and ZEB2, thereby inhibiting the mesenchymal phenotype. However, it is unclear how BBR regulates the relationship between TGF-β1, NF-κB p65 and related microRNAs to control colonic epithelial EMT.

In the current study, we explored the role of the crosstalk between TGF-β1/Smads and the NF-κB p65 pathway in the regulation of EMT by BBR in colon cancer cells via changes in microRNAs. We observed that TGF-β1 treatment caused NF-κB p65 activation and decreased the expression of Smad7 in colon cancer and normal colon epithelial cell lines. BBR downregulated the expression of p-Smad2, p-Smad3, NF-κB p65, and p-IκBα and upregulated Smad7. Interestingly, BBR-regulated miRNA expression patterns induced by TGF-β1 were completely different in normal colonic epithelium and colon cancer cells.

## 2 Materials and methods

### 2.1 Cell lines and culture conditions

The colorectal cancer cell line HT29 and the normal colonic epithelial cell line HCoEpiC were purchased from Guangzhou Ginio Biological Co., Ltd. (JNO-21409) and Guangzhou Ruile Biological Co., Ltd. (JNO-1787). HT29 cells and HCoEpiC cells were cultured separately in McCoy's 5A medium and MEM supplemented with 10 % foetal bovine serum (FBS) and 1 % penicillin‒streptomycin antibiotics, and the media was changed every 2–3 days. The cells were passaged at 80 % confluence and cultured in an incubator at 37 °C with 5 % CO_2_ and saturated humidity.

### 2.2 Primary reagents

McCOY's 5A (Guangzhou Noraxi Biotechnology Co., Ltd., PM150710), MEM (Procell, PM150410), PBS (HyClone, SH30256), Trypsin Enzyme (HyClone, SH30042.01), and foetal bovine serum (Gibco, 42G3279K) were used in the experiment. TGF-β1 was obtained from Puxin Biological Co., Ltd. (105-49), BBR was obtained from Sigma (B3251), SB431542 was obtained from Beyotime, Wuhan, China (P012809), and BAY 11–7082 was obtained from Beyotime, Wuhan, China (P012811). Rabbit antibodies against p-Smad2 (CST, 18338T), Smad3 (CST, 9520T), NF-κB p65 (Abcam, ab32536), p-IκBα (CST, 2859T), Smad7 (Abcam, ab216428), *E*-cadherin (Abcam, ab40772), and N-cadherin (Abcam, ab76011) were used in the study.

### 2.3 TGF-β1 induction and berberine treatment

HT29 and HCoEpiC cells were digested, resuspended, seeded into a 24-well plate and cultured overnight at 37 °C in a 5 % CO_2_ incubator. Different concentrations of TGF-β1 (0 ng/ml, 2 ng/ml, or 10 ng/ml) were then added to the culture medium. TGF-β1 stock solution was prepared by dissolving 10 μg of TGF-β1 in 1 ml of double distilled water to make a 10 μg/ml stock solution, which was then diluted 5000 times and 1000 times with culture medium to prepare a working solution containing 2 ng/ml TGF-β1. The cells were treated with TGF-β1 for 24 h, 48 h, or 72 h. The HT29 and HCoEpiC cells were divided into a TGF-β1 induction group and a berberine group. In the TGF-β1 induction group, 2 ng/ml TGF-β1 was added to the culture medium, and in the berberine induction group, 2 ng/ml TGF-β1 and 42 μmol/ml berberine were added to the culture medium, after which the cells were cultured for 72 h. At the end of the experiment, the cell morphology was observed under a microscope, and relevant indicators were measured.

### 2.4 Plasmid construction and transfection

The sequence design of NF-κB p65 and SMAD2 gene primers and the construction of plasmids were performed by Guangzhou Genio Biotechnology Co., LTD. NF-κB p65 gene serial number: NM_001165412.2 and andSMAD2 gene serial number: NM_001003652.4. The vectors were pCDNA3.1(+), pCDNA3.1(+)-SMAD2 and –NF–κB p65 were bacterial solution containing 20 % glycerol (1 ml), and plasmid 20 μl (2 μg). pCDNA3.1(+)-SMAD2 and –NF–κB p65 plasmids were transfected into HT29 and HCoEpiC cells (working concentration 50 μg/ml) according to the Lipofectamine™ 2000 operating instructions, and PCR was performed for verification 24 h after transfection.

### 2.5 Wound healing assay

Once the cells reached 85–90 % confluence, a scratch was made perpendicular to the cell culture plate with a 10 μl pipette tip. After washing three times with PBS, TGF-β1 and berberine were added (in serum-free culture medium), and images were acquired at 0, 24, 48, and 72 h. The area values were obtained by using ImageJ software. The migration rate was calculated as the ratio of the scratch width of the experimental group to that of the control group (0 h). The migration rate was calculated as (0 h scratch width - scratch width at the time point of treatment)/0 h scratch width.

### 2.6 Western blotting

The cells were treated with TGF-β1 or berberine and inoculated in 6-well plates. The plates were washed 2 times with ristocetin-induced platelet agglutination buffer (RIPA) plus 1 % phenylmethanesulfonylfluoride lysis solution being added to the cells. Centrifugation was performed at 12,000 rpm at 4 °C for 20 min to obtain the supernatant. A Bradford protein assay kit was used to determine the protein concentration. Sodium dodecyl sulfate‒polyacrylamide gel electrophoresis (SDS‒PAGE) was performed, and the proteins were transferred to membranes. The membranes were blocked with 5 % skim milk blocking buffer at room temperature for 2 h. The membranes were incubated with anti-E-cadherin, anti-N-cadherin, anti–NF–κB p65, anti-ph-IκBα, anti-Smad7, anti-ph-Smad2, anti-ph-Smad3 and anti-GAPDH antibodies (1:1000), as well as an HRP-labelled secondary antibody (1:10,000), for 1.5 h. The ECL exposure solution was a 1:1 mixture of liquid A:liquid B, and it was evenly applied to cover the entire membrane for 2 min, followed by exposure to detect the protein bands.

### 2.7 Real-time quantitative polymerase chain reaction (RT‒qPCR)

The relative levels of miRNA-1269a, miR-135b, miR-29a, miR-21, and miR-200a mRNA in HT29 and HCoEpiC cells were measured via RT‒qPCR according to the test procedure. RNA was extracted from the cells by using 1 ml of TRIzol reagent (TR118-500, MRC) and 200 μl of trichloromethane, followed by cDNA reverse transcription using M-MLV Reverse Transcriptase (M1705, Promega) and amplification using GoTaq® qPCR Master Mix (A6002, Promega). The sequences of the abovementioned miRNA primers were designed with Primer 5 (Table 1). The relative expression of each target gene was calculated as follows: 2^-△△Ct^ = 2^-[(△Ct)Test-(△Ct)Control]^.

**Table 1:** The sequences of the miRNA primers were designed by Primer 5.

Primer Name	5′ to 3′
hsa-miR-200a-3p Forward primer	GCGCTAACACTGTCTGGTAA
hsa-miR-200a-3p Reverse primer	GTCGTATCCAGTGCGTGTC
hsa-miR-1269a Forward primer	TAACTGGACTGAGCCGTGC
hsa-miR-1269a Reverse primer	GTCGTATCCAGTGCGTGTC
hsa-miR-21-5p Forward primer	CGCCTAGCTTATCAGACTGA
hsa-miR-21-5p Reverse primer	GTCGTATCCAGTGCGTGTC
hsa-miR-29a-3p Forward primer	CGGGTAGCACCATCTGAAAT
hsa-miR-29a-3p Reverse primer	GTCGTATCCAGTGCGTGTC
hsa-miR-135b-5p Forward primer	GCGGTATGGCTTTTCATTCCT
hsa-miR-135b-5p Reverse primer	GTCGTATCCAGTGCGTGTC
U6 Forward primer	CTCGCTTCGGCAGCACATATACT
U6 Forward primer	ACGCTTCACGAATTTGCGTGTC
hsa-miR-200a-3p RT primer	GTCGTATCCAGTGCGTGTCGTGGAGTCGGCAATTGCACTGGATACGACACATCG
hsa-miR-1269a RT primer	GTCGTATCCAGTGCGTGTCGTGGAGTCGGCAATTGCACTGGATACGACCCAGTA
hsa-miR-21-5p RT primer	GTCGTATCCAGTGCGTGTCGTGGAGTCGGCAATTGCACTGGATACGACTCAACA
hsa-miR-29a-3p RT primer	GTCGTATCCAGTGCGTGTCGTGGAGTCGGCAATTGCACTGGATACGACTAACCG
hsa-miR-135b-5p RT primer	GTCGTATCCAGTGCGTGTCGTGGAGTCGGCAATTGCACTGGATACGACTCACAT
U6 reverse transcription primer	AAAATATGGAACGCTTCACGAATTTG

### 2.8 Statistical analysis

All of the data were analysed by using the Solutions Statistical Package for the Social Sciences (SPSS) 23.0, and the results are presented as the mean (M) ± standard deviation (SD). Comparisons between multiple groups were performed by using one-way analysis of variance (ANOVA), and post hoc multiple comparisons were performed by using least significant difference (LSD) tests. *P* values < 0.05 were considered to indicate statistical significance.

## 3 Results

### 3.1 TGF-β1 significantly promoted EMT in both normal and cancerous colon epithelial cells

As shown by the microscopic morphological results (Fig. 1), 2 ng/ml TGF-β1 did not significantly affect the morphology of normal HCoEpiC or HT29 cancerous colon epithelial cells. However, after 72 h of treatment with 10 ng/ml TGF-β1, the morphology of both HCoEpiC and HT29 cells changed. Further analysis of epithelial marker protein expression demonstrated that TGF-β1 significantly promoted the downregulation of *E*-cadherin expression and the upregulation of N-cadherin expression in a concentration- and time-dependent manner (Table 2). The reduction in *E*-cadherin expression and increase in N-cadherin expression after 72 h of treatment with 10 ng/ml TGF-β1 were significantly greater than those after 24 h or 48 h of treatment with 2 ng/ml TGF-β1 (Fig. 2 and Supplementary Figs. S1–S2).

**Fig. 1:**
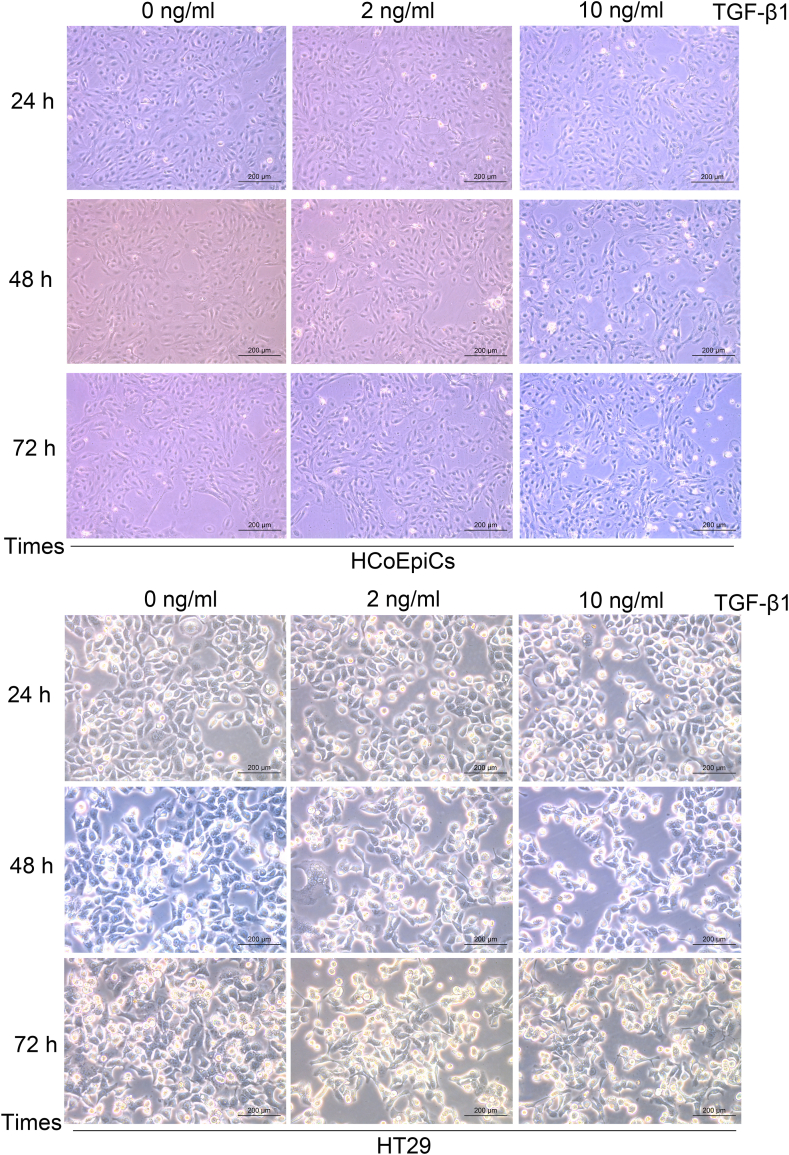
Morphological observations (40x10) of normal colonic epithelial HCoEpiC and colonic carcinoma HT29 cells treated with TGF-β1 at different concentrations (0 ng/ml, 2 ng/ml, or 10 ng/ml) for different durations (24 h, 48 h, or 72 h) (The scale bars = 200 μm).

**Table 2:** Comparison of relative expression of *E*-cadherin and N-cadherin protein (/GAPDH) in HT29 and HCoEpiC cells.

		HCoEpiC cells			HT29 cells		
		0 ng/ml	2 ng/ml	10 ng/ml	0 ng/ml	2 ng/ml	10 ng/ml
*E*-cadherin	24h	0.6801 ± 0.010	0.5293 ± 0.032	0.2133 ± 0.006	1.3887 ± 0.077	1.4319 ± 0.006	0.9948 ± 0.007
	48h	0.8258 ± 0.009	0.5292 ± 0.007	0.1719 ± 0.004	1.3203 ± 0.058	1.0326 ± 0.008	0.6424 ± 0.007
	72h	0.6021 ± 0.019	0.1255 ± 0.005	0.1402 ± 0.006	1.4659 ± 0.082	0.7714 ± 0.007	0.3167 ± 0.006
*P* value^a^		<0.001	<0.001	<0.001	0.127	<0.001	<0.001
N-cadherin	24h	0.1479 ± 0.005	0.2029 ± 0.003	0.5031 ± 0.006	0.1377 ± 0.006	0.1601 ± 0.006	0.4043 ± 0.005
	48h	0.1883 ± 0.004	0.5341 ± 0.006	0.9476 ± 0.019	0.1639 ± 0.006	0.3313 ± 0.007	0.6968 ± 0.006
	72h	0.2077 ± 0.008	0.7951 ± 0.025	1.0225 ± 0.015	0.1833 ± 0.006	0.8644 ± 0.008	1.5077 ± 0.006
*P* value^a^		<0.001	<0.001	<0.001	<0.001	<0.001	<0.001

a *P* value was the value obtained by one-way ANOVA, and LSD method was used for comparison between groups.

**Fig. 2:**
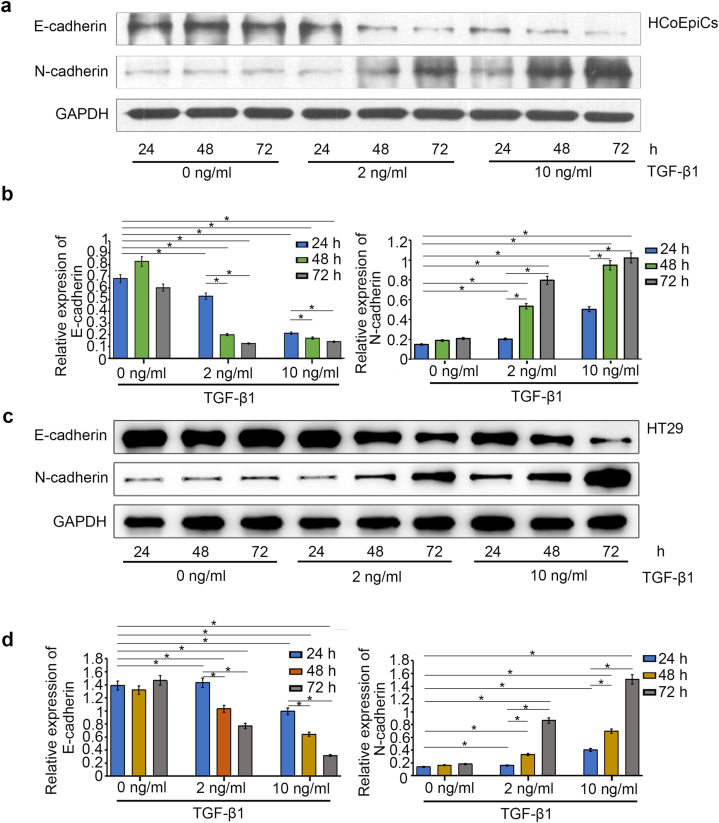
The expression of the EMT markers *E*-cadherin and N-cadherin was detected via western blotting. **a** Protein bands of HCoEpiC cells treated with different concentrations of TGF-β1. **b** Histogram of relative protein expression in HCoEpiC cells. **c** Protein bands of HT29 cells treated with TGF-β1 under different conditions. **d** Histogram of relative protein expression in HT29 cells. *P < 0.05 (The images of gels and blots has shown in Supplementary Figs. S1–S2).

### 3.2 Berberine (BBR) significantly inhibited the migration of normal and cancerous colon epithelial cells induced by TGF-β1

Previous experimental results have shown that TGF-β1 indeed significantly promoted EMT changes in normal and cancerous colon epithelial cells under in vitro conditions, and changes in EMT significantly enhanced the motility and migration ability of cells. Due to the fact that our previous studies confirmed that TGF-β1 enhanced the migration of colon epithelial cells in a concentration- and time-dependent manner, in this study, we investigated the effect of BBR on the migration of normal and cancerous colon epithelial cells induced by TGF-β1. We found (Fig. 3 and Table 3) that BBR significantly suppressed the migration of HCoEpiC and HT29 cells stimulated by TGF-β1. Further observation of the changes in EMT markers demonstrated that BBR significantly promoted the expression of *E*-cadherin in cells while also inhibiting the expression of N-cadherin (Fig. 4a and Supplementary Fig. S3), thus suggesting that BBR may mediate cell migration by inhibiting the EMT of cells induced by TGF-β1.

**Fig. 3:**
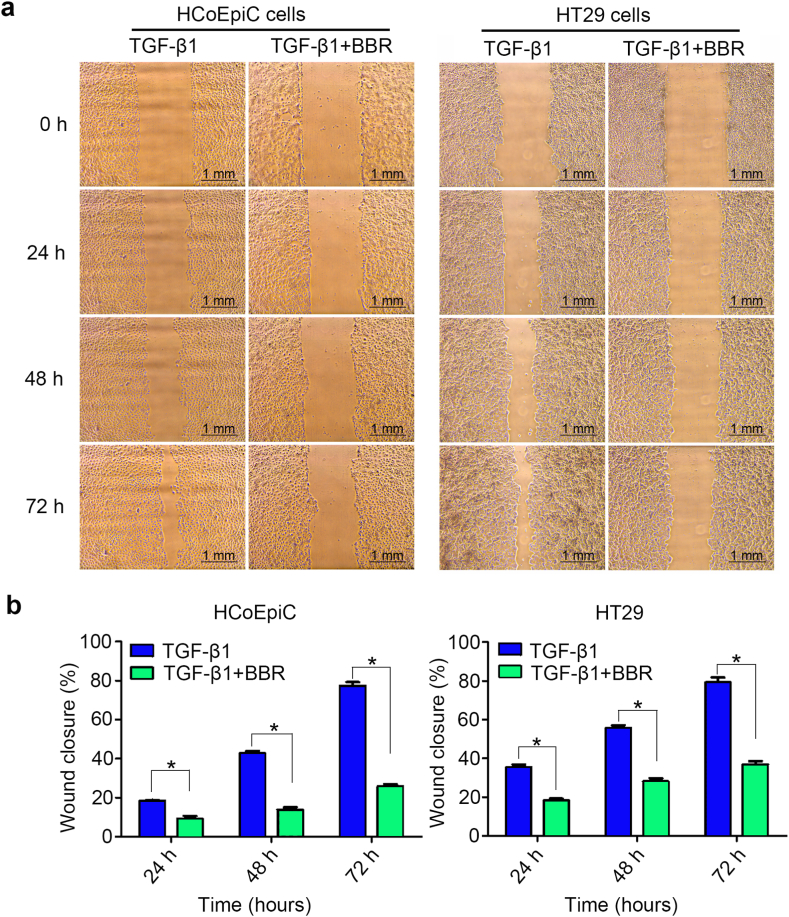
Comparison of cell mobility between different treatment groups. **a** Morphological observations of wound healing in HCoEpiC and HT29 cells treated with TGF-β1 and BBR (4x10) (The scale bars = 1.0 mm). **b** Comparison of cell mobility between the two groups. The 0 h mobility of the TGF-β1 and BBR groups was 0. *P < 0.05.

**Table 3:** Cell mobility rate between different treatment groups.

Cells	Group	0 h	24 h	48 h	72 h
HCoEpiC	TGF-β1	0	18.298 ± 1.114	42.723 ± 2.728	77.404 ± 4.354
	TGF-β1+BBR	0	9.224 ± 3.38	13.831 ± 2.873	25.748 ± 2.490
*P* value		–	<0.001	<0.001	<0.001
HT29	TGF-β1	0	35.368 ± 3.534	55.527 ± 3.674	79.232 ± 6.298
	TGF-β1+BBR	0	18.185 ± 2.554	28.128 ± 3.875	36.895 ± 3.993
*P* value		–	<0.001	<0.001	<0.001

The mobility rate was calculated by Area/height, namely, rate= (0 h group - treatment group)/0 h group × 100 %).

**Fig. 4:**
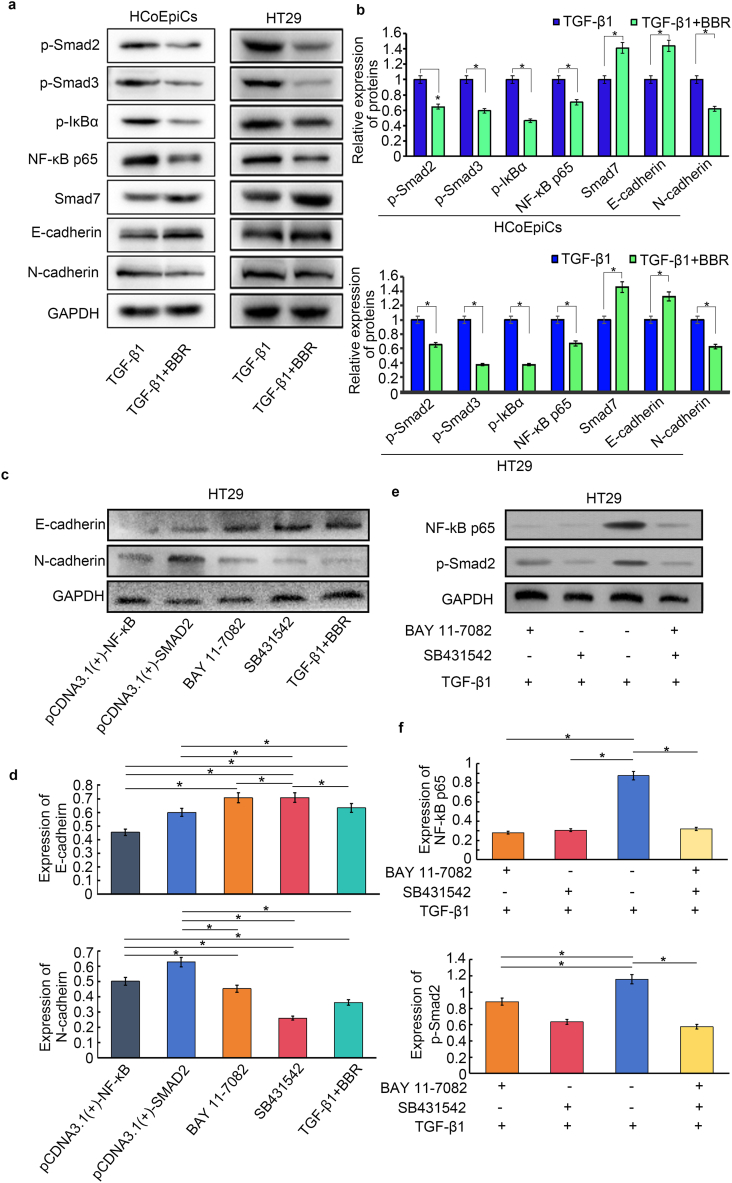
Detection and comparison of related proteins in cells. **a** Comparison of Smads, NF-κB p65 pathway major molecules and EMT marker protein bands of HCoEpiC and HT29 cells treated with TGF-β1 and BBR for 72 h (Supplementary Fig. S3). **b** Histogram of Smads, NF-κB p65 pathway major molecules and EMT marker protein expressions of HCoEpiC and HT29 cells. **c** Expression of *E*-cadherin and N-cadherin in HT29 cells treated with inhibitors of the TGF-β1/Smad and NF-κB p65 pathways and overexpression of SMAD2 (pCDNA3.1[+]-SMAD2) and NF-κB p65 (pCDNA3.1[+]–NF–κB) for 72 h (Supplementary Fig. S4). **d** Comparison of the protein expression of *E*-cadherin and N-cadherin in HT29 cells after different treatments. **e** Protein expression bands of NF-κB p65 and p-Smad2 in HT29 cells treated with BAY 11–7082 and SB431542 for 72 h (Supplementary Fig. S4). **f** Comparison histograms of NF-κB p65 and p-Smad2 expression in HT29 cells (*P < 0.05).

### 3.3 Berberine regulated the TGF-β1/Smad and NF-κB p65 pathways in normal and cancerous colon epithelial cells

TGF-β1 can induce EMT changes in epithelial cells through the classic Smad pathway, and the regulation of EMT by BBR may influence the TGF-β1/Smad pathway. As shown in Fig. 4a and b, BBR significantly reduced the expression levels of p-Smad2 and p-Smad3 induced by TGF-β1 in HCoEpiC and HT29 cells but also increased the expression of Smad7. Furthermore, we also found that compared to those in the TGF-β1 treatment group, the expression of NF-κB p65 and p-IκBα in the BBR treatment group was significantly lower, thus indicating that BBR not only inhibited TGF-β1-induced Smad pathway activation but also suppressed activated NF-κB signalling. When considering that the changes in the TGF-β1/Smad and NF-κB p65 pathways in HCoEpiC and HT29 cells were similar, after overexpressing NF-κB p65 and Smad2 in HT29 cells (by pCDNA3.1(+)–NF–κB p65 and -SMAD2), we found a decrease in the expression of *E*-cadherin and a relative increase in the expression of N-cadherin. In contrast, the effects of the TGF-β1/Smad pathway inhibitor SB431542 and the NF-κB p65 pathway inhibitor BAY 11–7082 were similar to the effects of BBR, with both showing an increase in *E*-cadherin expression and a decrease in N-cadherin expression (Fig. 4c and d and Supplementary Fig. S4).

To further explore the relationship between the TGF-β1/Smad pathway and the NF-κB p65 pathway in HT29 cells, we observed the expression of NF-κB p65 and p-Smad2 in cells treated with SB431542 and BAY 11–7082, respectively (Fig. 4e and f and Supplementary Fig. S4). We found that compared to those in the group treated with only TGF-β1, the expression of NF-κB p65 and p-Smad2 decreased with BAY 11–7082 treatment (*P* < 0.05). In cells treated with SB431542, the expression of NF-κB p65 significantly decreased (compared to that in the TGF-β1 treatment group, *P* < 0.05). However, we found that the expression of p-Smad2 in cells treated with BAY 11–7082 was greater than that in the SB431542 treatment group. The effects of combining BAY 11–7082 and 431542 were similar to those of BAY 11–7082 alone.

### 3.4 Berberine mediated the expression pattern of microRNAs involved in TGF-β1-induced EMT in normal colon epithelial cells, but this pattern differed from that in cancerous colon epithelial cells

**Fig. 5:**
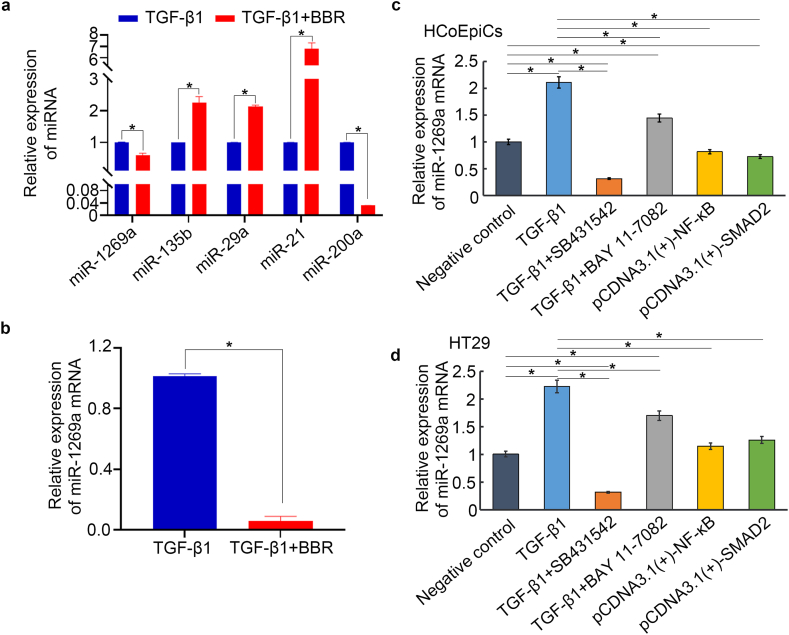
Expression of miRNAs in HCoEpiC and HT29 cells. **a** Expression of miRNAs in HCoEpiC cells treated with TGF-β1 and BBR. **b** Changes in the expression of miRNA-1269a in HT29 cells (the detection results for other miRNAs were negative; therefore, only the results for miRNA-1269a are shown). **c** Expression of miRNA-1269a mRNA in HCoEpiC cells treated with SB431542, BAY 11–7082, pcDNA3.1(+)-SMAD2 and pcDNA3.1(+)–NF–κB. **d** Changes in the miRNA-1269a mRNA level in HT29 cells treated with SB431542 and BAY 11–7082 and overexpression of SMAD2 and NF-κB p65. The negative control group did not receive any treatment. *P < 0.05.

The abovementioned results showed that BBR can regulate the TGF-β1-induced Smad pathway and NF-κB signalling. Recent research has demonstrated that regulatory relationships exist between the TGF-β1/Smad or NF-κB pathways and microRNAs [18,19]. In our study, we found that BBR significantly regulated the miRNAs induced by TGF-β1 in HCoEpiC and HT29 cells, but the patterns were different. Compared to those in the TGF-β1 treatment group, BBR significantly reduced the expression levels of miR-1269a and miR-200a induced by TGF-β1 in HCoEpiC cells while increasing the expression of miR-135b, miR-29a, and miR-21, with the increase in miR-21 being the most significant (Fig. 5a). However, in TGF-β1-induced HT29 cells, we detected that BBR significantly decreased the expression of miR-1269a in only HT29 cells (Fig. 5b); however, after repeated testing, we found that the expression of miR-200a, miR-135b, miR-29a, and miR-21 was negative, which may suggest that miR-200a, miR-135b, miR-29a, and miR-21 may not be expressed in HT29 cells.

Afterwards, we observed the regulatory effects of the TGF-β1 and NF-κB pathways on miR-1269a in HCoEpiC and HT29 cells. We used BAY 11–7082 and 431542 in both cell lines and overexpressed NF-κB p65 and SMAD2. The results showed (Fig. 5c and d) that, compared with the negative control group (without treatment), SB431542 significantly reduced the mRNA level of miR-1269a in HCoEpiC and HT29 cells (*P* < 0.05), while BAY 11–7082 increased its expression (*P* < 0.05); however, both were lower than those in the group treated only with TGF-β1 (*P* < 0.05). Conversely, after overexpressing NF-κB p65 and SMAD2, the mRNA level of miR-1269a in both cell types increased but was significantly lower than that in the TGF-β1 treatment group (*P* < 0.05).

## 4 Discussion

TGF-β1, which is a known inducer and promoter of EMT, induces the expression of EMT-related genes through the classical Smad pathway. These genes include EMT transcription factors such as Snail, Slug, and Twist, which downregulate epithelial markers (such as *E*-cadherin) and upregulate mesenchymal markers (such as N-cadherin and Vimentin). EMT promotes cell growth, invasion and metastasis. Therefore, our current study was consistent with previous results [20], thus suggesting that TGF-β1 can promote EMT changes in both normal and cancerous colonic epithelial cells under in vitro conditions. This finding also indicates that the induction of EMT by TGF-β1 is not significantly associated with the intrinsic benign or malignant properties of the cells. Furthermore, this induction effect was positively correlated with both time and concentration. The longer the epithelial cells were exposed to TGF-β1 and the higher the concentration, the greater the ability of the epithelial cells to undergo EMT. This may also explain why, in the TGF-β1-rich tumour microenvironment, many stromal cells undergo EMT, thus promoting the accumulation of tumour-associated fibroblast-like cells, which correspondingly promotes more cells to undergo EMT by secreting TGF-β1. Therefore, a number of drugs such as small molecule preparations or antibodies targeting TGF-β1 signalling have been developed for cancer treatment.

Berberine (BBR) has been widely studied for its numerous pharmacological effects. Similar to our previous findings [20], BBR can significantly inhibit the EMT and migration of epithelial cells induced by TGF-β1, which may be related to its regulation of Smad pathway activity. This effect is due to the fact that we observed that the expression of p-Smad2/3 in cells treated with BBR was significantly lower than that in the TGF-β1 treatment group; moreover, BBR promoted the upregulation of Smad7 expression.

In fact, the TGF-β1/Smad pathway often does not act alone. Recent studies have demonstrated that TGF-β1 can either synergize with or antagonize NF-κB signalling [21,22], which is dependent on the cellular context and stimulus. For instance, in the context of inflammation, TGF-β1 has been shown to inhibit NF-κB activation, thereby exerting anti-inflammatory effects. This effect is achieved through TGF-β1-induced expression of Smad7, which interferes with the activation of IκB kinase (IKK), which is a key regulator of NF-κB [23]. Conversely, under certain conditions, TGF-β1 can enhance NF-κB signalling, thus promoting cell survival and proliferation. This positive interaction often occurs through the TGF-β1-mediated downregulation of IκB, which is an inhibitor of NF-κB, thus leading to increased nuclear translocation of NF-κB and transcriptional activation of target genes.

**Fig. 6:**
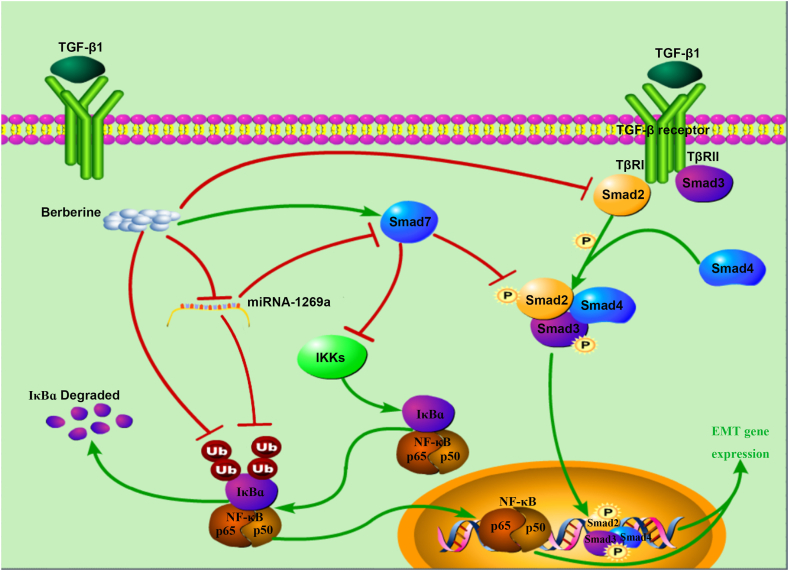
Schematic of the results of this study suggesting the possible pharmacological targets of berberine. As an inhibitory regulator of the TGF-β1/Smad pathway, Smad7 is inhibited during TGF-β1-induced colonic epithelial EMT. Inactivated Smad7 leads to the activation of IKK, which promotes IκBα activation and ubiquitination degradation, thus leading to the activation of NF-κB p65. Berberine can not only inhibit the phosphorylation of Smad2/3 and the ubiquitination-mediated degradation of IκBα but also promote the upregulation of Smad7 expression and reverse the inhibition of TGF-β1/Smad and NF-κB pathway activity. Berberine inhibits the expression of miR-1269a to promote Smad7 transcription, and miR-1269a triggers the ubiquitination and degradation of IκBα to promote the activation of NF-κB p65. Berberine suppresses the transcription of miR-1269a, thereby inhibiting the translocation and expression of NF-κB p65. The green arrows represent promotion or activation, and the red flat heads represent inhibition or deactivation. P: phosphorylation. Ub: ubiquitination.

Alternatively, the dichotomous relationship between TGF-β1 and NF-κB is further complicated by the fact that NF-κB can also regulate TGF-β1 signalling [24]. NF-κB has been reported to induce the expression of TGF-β1, thus creating a feedback loop that modulates the extent and duration of the response to TGF-β1. Additionally, NF-κB can influence the outcome of TGF-β1 signalling by regulating the expression of Smad proteins, thus further highlighting the intricate interplay between these two pathways. The results of this study showed that BBR not only inhibited the activity of the TGF-β1/Smad pathway but also suppressed the expression of p-IκBα and NF-κB p65, thus indicating that BBR may have a regulatory effect on both the TGF-β1/Smad and NF-κB pathways (Fig. 6). Therefore, we believe that whether BBR can have a synergistic effect with TGF-β1 inhibitors in the future clinic treatment of cancer, which needs further in-depth research.

Previous studies have demonstrated that BBR inhibits the activation of the NF-κB pathway by blocking the degradation of IκBα, which is an inhibitor of NF-κB [25]. By preventing IκBα degradation, BBR maintains NF-κB in an inactive state in the cytoplasm, thereby reducing the transcription of NF-κB target genes. By inhibiting NF-κB activation, BBR can induce apoptosis in cancer cells and inhibit tumour growth [26]. The mechanism involves not only the suppression of antiapoptotic genes regulated by NF-κB but also the modulation of cell cycle regulators and proapoptotic proteins. In addition to its direct inhibition of NF-κB, BBR has been shown to interfere with various upstream signalling pathways that lead to NF-κB activation, including the TLR4/MyD88 and JAK/STAT pathways [27,28]. This broad-spectrum inhibition amplifies its anticancer effects, thus making berberine a compound of interest for therapeutic development.

However, it is not yet clear how the TGF-β1/Smad and NF-κB pathways specifically contribute to the inhibition of EMT by BBR. In this study, we constructed overexpression vectors for SMAD2 and NF-κB p65 and transfected them into HT29 cells by adding the TGF-β1/Smad pathway inhibitor SB431542 and the NF-κB pathway inhibitor BAY 11–7082 to the cell culture medium. The results showed that compared to cells treated with SB431542, BAY 11–7082, and BBR, cells overexpressing SMAD2 and NF-κB p65 exhibited a certain degree of suppression of *E*-cadherin expression, whereas N-cadherin expression was upregulated to varying extents. However, the expression of N-cadherin was more pronounced in cells overexpressing SMAD2 than in those overexpressing NF-κB p65. These findings suggest that the TGF-β1/Smad pathway may play a major role in EMT changes in colonic epithelial cells. It is also possible that during the EMT process in colorectal cancer epithelial cells, TGF-β1/Smads induces the activation of the NF-κB p65 pathway, which then jointly promotes EMT.

Therefore, to further observe the correlation between the TGF-β1/Smad and NF-κB p65 pathways, we detected changes in NF-κB p65 and p-Smad2 expression after blocking the TGF-β1/Smad and NF-κB p65 pathways. The results showed that after inhibiting the TGF-β1/Smad pathway with SB431542, the expression of NF-κB p65 significantly decreased. Conversely, after blocking the NF-κB pathway with BAY 11–7082, the p-Smad2 level decreased, but it was greater than that in cells treated with SB431542 but lower than that in the TGF-β1 treatment group. These findings suggest that TGF-β1/Smads may have a primary regulatory effect on the NF-κB p65 pathway and that BBR may primarily inhibit the expression of NF-κB p65 by suppressing the TGF-β1/Smad pathway, thereby regulating the EMT of colorectal cancer cells.

MicroRNAs (miRNAs) are short noncoding RNA molecules that regulate gene expression and play a role in a variety of biological processes, including EMT, by binding to the 3′ untranslated region (3′UTR) of the target mRNA, thus causing its degradation or translation inhibition. Studies have shown that the miR-200 family maintains epithelial status and inhibits EMT by directly targeting ZEB1 and ZEB2 transcription suppressors [29]. In contrast, ZEB1 and ZEB2 inhibit the expression of the miR-200 family, thus creating a feedback loop that promotes EMT and cancer cell aggressiveness [30]. In addition, different miRNAs can influence the EMT process by regulating key molecules in the TGF-β, Wnt/β-catenin, Notch and other signalling pathways. Therefore, the EMT process can be reversed by regulating the expression of microRNAs. Our results showed that BBR can regulate the expression profile of multiple miRNAs involved in the TGF-β1-induced EMT of normal colonic epithelial HCoEpiC cells by significantly reducing the expression of miR-1269a and miR-200a while increasing the expression of miR-135b, miR-29a and miR-21. However, we found that in the TGF-β1-induced EMT of colon cancer HT29 cells, only miR-1269a was expressed among the abovementioned miRNAs, and other miRNAs were not expressed. As in HCoEpiC cells, BBR also significantly reduced the TGF-β1-induced upregulation of miR-1269a expression in HT29 cells. Therefore, we speculate that miR-1269a may have a similar mechanism in the EMT of normal and cancerous colon epithelial cells.

Several recent studies have shown that microRNA-1269a can promote cancer occurrence and progression [[31], [32], [33]]. Afterwards, we observed changes in the expression of miR-1269a after the overexpression of Smad2 and NF-κB p65 and the inhibition of the TGF-β1/Smad and NF-κB p65 pathways. Compared with the negative control group, SB431542 treatment significantly reduced the mRNA level of miR-1269a in HCoEpiC and HT29 cells (*P* < 0.05), whereas BAY 11–7082 treatment increased its expression (*P* < 0.05). However, these parameters were lower in both groups than in the group treated with TGF-β1 alone (*P* < 0.05). Conversely, the mRNA levels of miR-1269a were increased after the overexpression of NF-κB p65 and SMAD2 but were significantly lower than those in the TGF-β1 treatment group (*P* < 0.05). A possible explanation for these results is that miR-1269a may act as an oncogene that synergistically promotes the TGF-β1/Smad and NF-κB p65 pathways associated with Smad7, and miR-1269a may be partially reversely regulated by the TGF-β1/Smad and NF-κB p65 pathways, which are related to the regulation of Smad7 (Fig. 6).

In conclusion, BBR can significantly inhibit TGF-β1-induced EMT in normal and cancerous colon epithelial cells. The mechanism involves the inhibitory regulation of the TGF-β1/Smad and NF-κB p65 pathways. TGF-β1/Smads can promote the NF-κB p65 pathway and may play a major role in the EMT process. During BBR intervention in TGF-β1-induced EMT, the expression pattern and mechanism of microRNAs in normal and cancerous cells may differ. TGF-β1/Smad and NF-κB p65 may be common targets of miR-1269a and may also partially regulate the expression of miR-1269a.

## Ethics declarations

Any ethics related to humans and animals were not involved in this study.

## Data availability statement

The data used to support the findings of this study are available from the corresponding authors upon request.

## CRediT authorship contribution statement

**chao Huang:** Writing – original draft. **Haosheng liu:** Software, Investigation. **Yidian Yang:** Visualization, Software. **Yue He:** Data curation. **Weizeng shen:** Supervision.

## Declaration of competing interest

The authors declare that they have no known competing financial interests or personal relationships that could have appeared to influence the work reported in this paper.

## Figures and Tables

**figs1:**
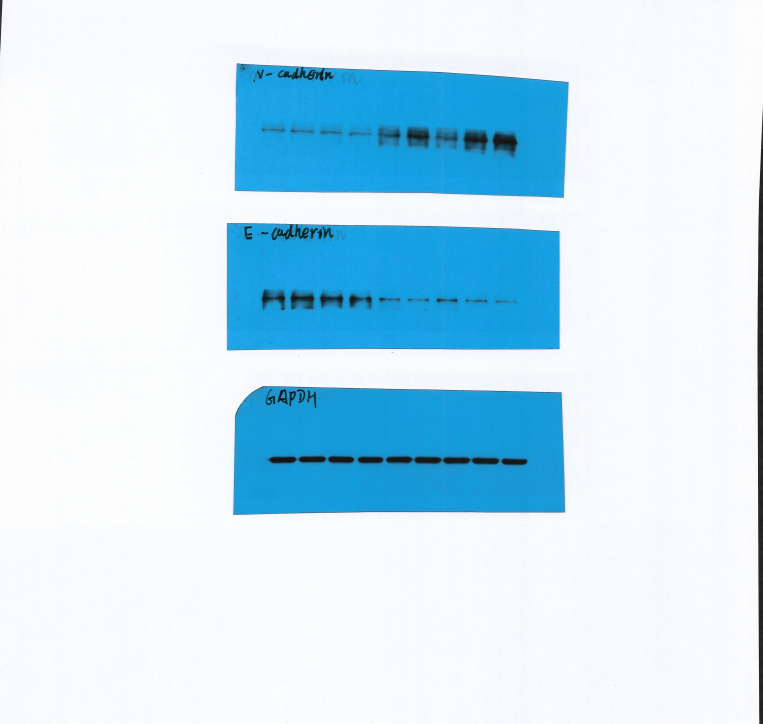


**figs2:**
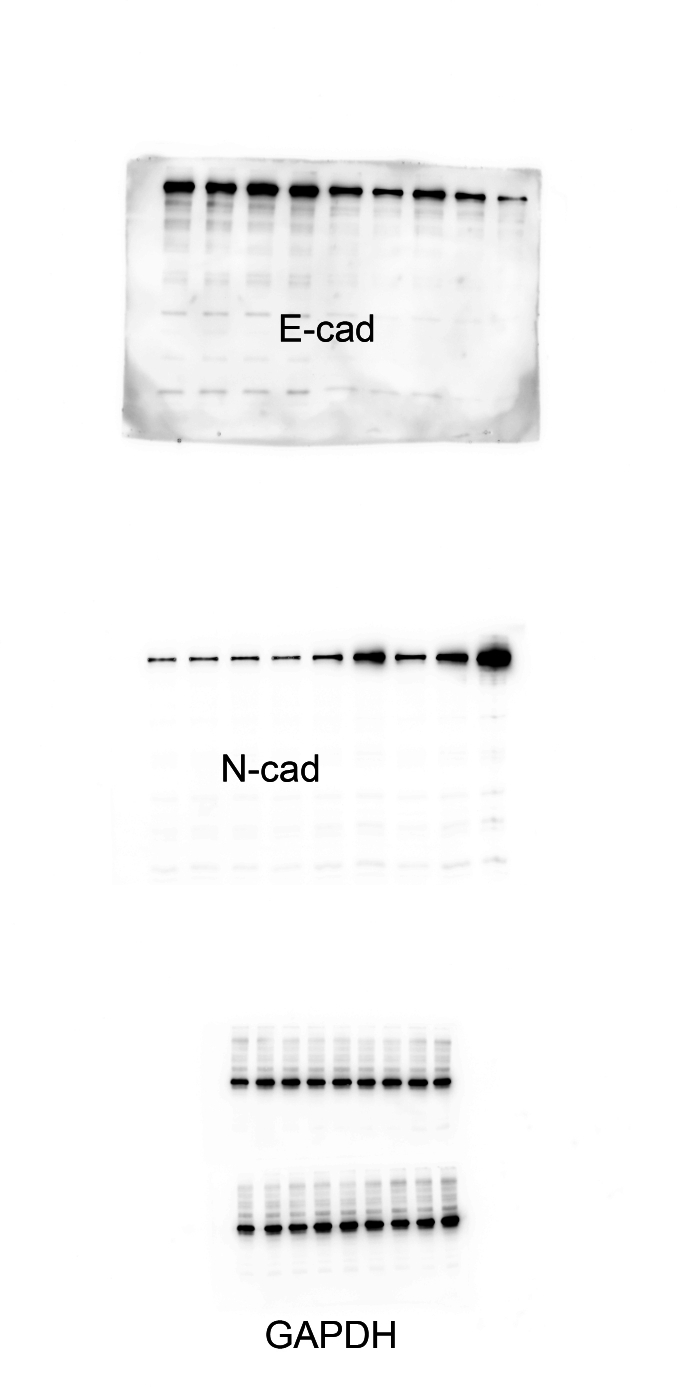


**figs3:**
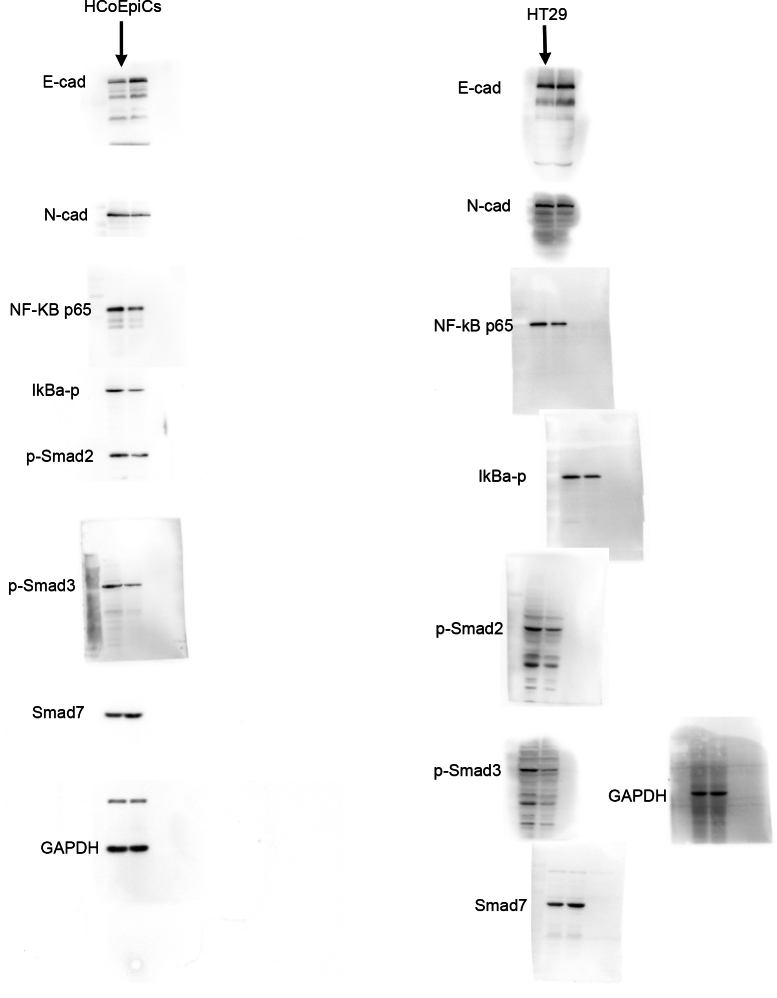


**figs4:**